# Norovirus-like VP1 particles exhibit isolate dependent stability profiles

**DOI:** 10.1088/1361-648X/aaa43b

**Published:** 2018-01-18

**Authors:** Ronja Pogan, Carola Schneider, Rudolph Reimer, Grant Hansman, Charlotte Uetrecht

**Affiliations:** 1 Heinrich Pette Institute, Leibniz Institute for Experimental Virology, Hamburg, Germanycharlotte.uetrecht@xfel.eu; 2 Department of Infectious Diseases, Virology, Heidelberg University, Heidelberg, Germany; 3 Schaller Research Group at the University of Heidelberg and the DKFZ, Heidelberg, Germany; 4 European XFEL, Schenefeld, Germany

**Keywords:** virus assembly, native mass spectrometry, structural virology, capsid stability, VLP size

## Abstract

Noroviruses are the main cause of viral gastroenteritis with new variants emerging frequently. There are three norovirus genogroups infecting humans. These genogroups are divided based on the sequence of their major capsid protein, which is able to form virus-like particles (VLPs) when expressed recombinantly. VLPs of the prototypical GI.1 Norwalk virus are known to disassemble into specific capsid protein oligomers upon alkaline treatment. Here, native mass spectrometry and electron microscopy on variants of GI.1 and of GII.17 were performed, revealing differences in terms of stability between these groups. Beyond that, these experiments indicate differences even between variants within a genotype. The capsid stability was monitored in different ammonium acetate solutions varying both in ionic strength and pH. The investigated GI.1 West Chester isolate showed comparable disassembly profiles to the previously studied GI.1 Norwalk virus isolate. However, differences were observed with the West Chester being more sensitive to alkaline pH. In stark contrast to that, capsids of the variant belonging to the currently prevalent genogroup GII were stable in all tested conditions. Both variants formed smaller capsid particles already at neutral pH. Certain amino acid substitutions in the S domain of West Chester relative to the Norwalk virus potentially result in the formation of these *T*  =  1 capsids.

## 1. Introduction

Human noroviruses cause the majority of nonbacterial gastroenteritis outbreaks worldwide (Ahmed *et al* 2014). Symptoms include fever, vomiting, and diarrhea that usually last for several days (Kaplan *et al* 1982). In addition to the surface of fecal contaminated food, noroviruses can be found in environmental samples such as sewage and spread through person-to-person contact (Kaplan *et al* 1982, Hedberg and Osterholm 1993, Ozawa *et al* 2007). A small number of particles of this pathogen is sufficient for infection. Severe disease is mostly observed in the elderly, children and immunocompromised people (Teunis *et al* 2008).

Noroviruses belong to the *Caliciviridae* family and are non-enveloped, single-stranded, positive-sense ribonucleic acid (RNA) viruses. Human norovirus genomes are 7.4–7.7 kb in length and have three open reading frames (ORFs). The non-structural proteins, such as the RNA dependent polymerase (RdRp), are encoded by ORF1, while the structural proteins are encoded by ORF2 (VP1) and ORF3 (VP2) (Xi *et al* 1990, Jiang *et al* 1993). The major capsid protein VP1 forms dimers, which assemble into the viral capsid with *T*  =  3 icosahedral symmetry of approximately 36–42 nm diameter. VP2 is considered as minor capsid protein and carries multiple functions, which are still not completely deciphered (Vongpunsawad *et al* 2013). VP1 can be divided into a shell (S) domain and a protruding (P) domain, which is further subdivided into P1 and P2. The S domain forms a scaffold surrounding the viral RNA with a diameter of 30 nm, whereas the genetically more variable P domain builds the viral spikes and facilitates cell attachment (Prasad *et al* 1994, 1999). Research on human noroviruses has been limited by the lack of a robust cell culture system. Nevertheless, production of virus-like VP1 particles (VLPs) show comparable characteristics to the native virion (Jiang *et al* 1992). Electron microscopy (EM) and x-ray crystallography studies have deepened the understanding of capsid assembly and interactions with potential receptors like human histo-blood group antigens (Harrington *et al* 2002, Tan and Jiang 2005).

Native mass spectrometry (MS) has further elucidated these processes (Shoemaker *et al* 2010, Uetrecht and Heck 2011, Mallagaray *et al* 2015). Here, intact non-covalent protein complexes are transferred into the gas phase from an aqueous solution of a volatile buffer surrogate, such as ammonium acetate, via nano-electrospray ionization (ESI). All coexisting oligomers can be detected with high sensitivity, allowing to monitor assembly processes. Recently, charge detection MS has been introduced, enabling simultaneous charge and mass-to-charge (*m*/*z*) ratio determination and therefore mass assignment of single ions (Keifer *et al* 2017). VLPs can also be analyzed by mobility-based approaches (Weiss *et al* 2015). However, ESI with time-of-flight (ToF) detectors are mostly employed, which rely on charge state resolution for exact mass assignment of *m*/*z* signals.

Remarkably, Shoemaker *et al* demonstrated the alkaline and ionic strength sensitivity of norovirus VLPs, and showed that capsid disassembly was reversible (Shoemaker *et al* 2010). VLPs completely disassembled at low ionic strength and alkaline pH, whereas smaller particles formed at high ionic strength in alkaline conditions. Most importantly, they showed that small VLPs, which were previously observed in EM under alkaline conditions, corresponded to *T*  =  1 capsids. The dynamic behavior of VP1 upon changing pH may have implications for the infection process as the virus passes through very different environments in the intestinal tract. Using ion mobility, pH dependent intermediates were shown to be consistent with partial capsid structures and an assembly pathway was proposed (Uetrecht *et al* 2011). These findings confirmed previous studies, which combined EM with spectroscopic techniques like circular dichroism and high resolution second-derivative UV absorption spectroscopy (Ausar *et al* 2006).

Importantly, MS studies focused on the prototypical Norwalk virus first isolated in 1972 in Ohio (Kapikian *et al* 1972), disregarding the emergence of new variants, which could exhibit altered assembly behavior. Noroviruses can be divided into at least seven genogroups (GI-GVII), with GI, GII, and GIV causing infections in human. Each genogroup can be further subdivided into numerous genotypes based on the capsid amino acid sequences (Kageyama *et al* 2004, Hansman *et al* 2006). The prototypical Norwalk virus (GI.1 Norwalk) is rarely found to cause outbreaks nowadays. It is replaced by strains of the genogroup II, where GII.4 noroviruses have been prevalent (Eden *et al* 2013). A reemerging genotype of this genogroup, GII.17, seems to become a major threat nowadays in East Asia. First isolated in 1978, they were the cause of several outbreaks in Asia in 2014 to 2015 (Chan *et al* 2015, Lee *et al* 2015, Zhang *et al* 2015, Singh *et al* 2016).

Here, GI.1 West Chester noroviruses closely related to and isolated three decades after the Norwalk strain and a newly emerging GII.17 variant isolated 2015 in Kawasaki, Japan, are compared. The VLP stability in different pH and ionic strength conditions of these genotypes was examined using native MS and negative stain EM. Surprisingly, the outbreak-causing isolate showed a much stronger resistance to pH. Furthermore, both variants form smaller *T*  =  1 capsids at neutral pH, which points to amino acid substitutions potentially influencing the particle size. Differences between variants in terms of mass and stability hint towards new approaches for virus identification.

## 2. Experimental details

### 2.1. VLP production and preparation

In order to produce norovirus VLPs, recombinant VP1 protein was expressed in insect cells as described previously (Hansman *et al* 2005, 2007). Genebank accession numbers are AY602016.1 and Kawasaki308 for the GI.1 and GII.17 variant, respectively. Briefly, recombinant VP1 bacmid was transfected into *Spodoptera frugiperda* (Sf9) cells using Effectene. After incubation for five days at 27 °C, the cell culture medium was clarified by low-speed centrifugation. The supernatant containing the baculovirus was used to infect high five (H5) insect cells for 6 d at 27 °C. To separate secreted VLPs from the cell medium and cells, the solution was centrifuged at low speed and then concentrated by ultracentrifugation at 35 000 rpm for 2 h at 4 °C. VLPs were purified using a caesium chloride (CsCl) equilibrium gradient ultracentrifugation at 35 000 rpm for 18 h at 4 °C and then stored in phosphate buffered saline at 4 °C at a concentration of 3–5 mg ml^−l^.

Prior to MS and EM analysis, the VLPs were exchanged into 50 and 250 mM ammonium acetate solutions at different pH using Vivaspin 500 centrifugal concentrators (10 000 MWCO, Sartorius, Göttingen, Germany). Zeba micro spin^™^ desalting columns 0.5 ml (7000 MWCO, Thermo Fisher Scientific, Massachusetts, USA) were used if protein yields were too low for analysis using Vivaspin.

### 2.2. Mass spectrometry

Native MS measurements on VLPs were performed on a Q-Tof 2 instrument (Waters, Manchester, UK and MS Vision, Almere, the Netherlands) with modifications enabling high mass experiments (van den Heuvel *et al* 2006). All spectra were recorded in positive ion mode. Generally, samples were measured at a VP1 concentration of 10 *µ*M. Ions were introduced via a nano-ESI source into the vacuum at a source pressure of 10 mbar using both premade (Waters, Manchester, UK) and handmade capillaries. For the in-house production, borosilicate glass tubes (inner diameter 0.68 mm, outer diameter 1.2 mm with filament; World Precision Instruments, Sarasota, USA) were pulled using a two-step program in a micropipette puller (Sutter Instruments, Novato, USA) with a squared box filament (2.5  ×  2.5 mm). The capillaries were gold-coated using a sputter coater (Quorum Technologies Ltd., East Sussex, UK, 40 mA, 200 s, tooling factor of 2.3 and end bleed vacuum of 8  ×  10^−2^ mbar argon) and opened directly on the sample cone of the mass spectrometer. Voltages of 1.55–1.65 kV and 145–155 V were applied to the capillary and cone, respectively. Xenon was used as a collision gas at a pressure of 2.0  ×  10^−2^ mbar to improve the transmission of large ions (Lorenzen *et al* 2007). Starting from 10 V, collision energies were increased up to 200 V in steps of 25 V. The presented spectra were generally recorded at 50 V collision energy. MS profile and pusher settings were kept constant for all measurements. A caesium iodide spectrum was recorded the same day in order to test the performance of the instrument and to calibrate spectra where necessary. Analysis was performed using MassLynx^™^ (Waters, Manchester, UK). In order to generate intensity fraction plots, signals were accumulated over 100 s and in a respective *m*/*z* range all signals belonging to the broad ion distributions of a given oligomer were summed. The range was kept constant for both variants to simplify comparison, although ion distribution widths varied. Tables S2 and S3 (stacks.iop.org/JPhysCM/30/064006/mmedia) list respective *m*/*z* ranges that correspond to different VP1 oligomers and their intensity fractions.

### 2.3. Electron microscopy

After buffer exchange to ammonium acetate solutions, the norovirus VLPs were stored at 4 °C. The samples were adsorbed onto glow discharge-activated carbon coated grids (Science Services, Munich, Germany) after storage in ammonium acetate solution. The sample coated grids were washed three times with distilled water, following a negative staining with 1% uranyl acetate. Images were acquired using the FEI Tecnai^™^ G2 transmission electron microscope and the wide angle Veleta CCD camera (FEI, Thermo Fisher Scientific, USA and Olympus, Tokyo, Japan) at 80 kV.

## 3. Results and discussion

### 3.1. Altered capsid stability and size distribution in GI.1 isolates

**Figure 1.**
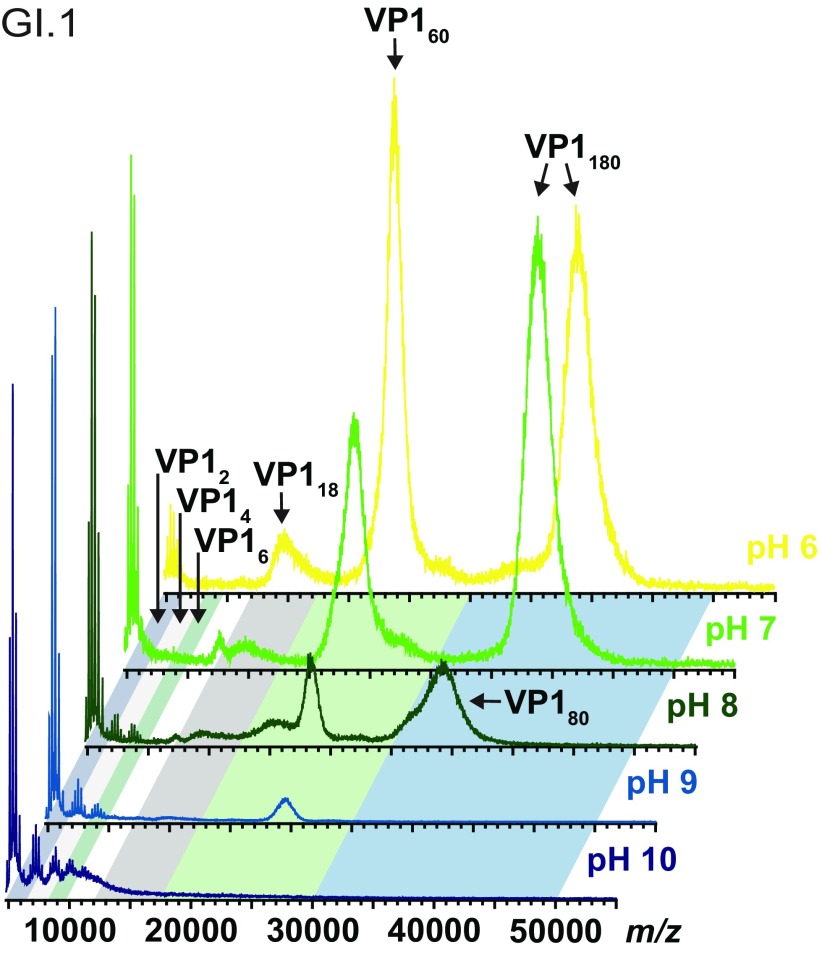

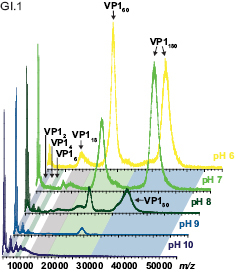

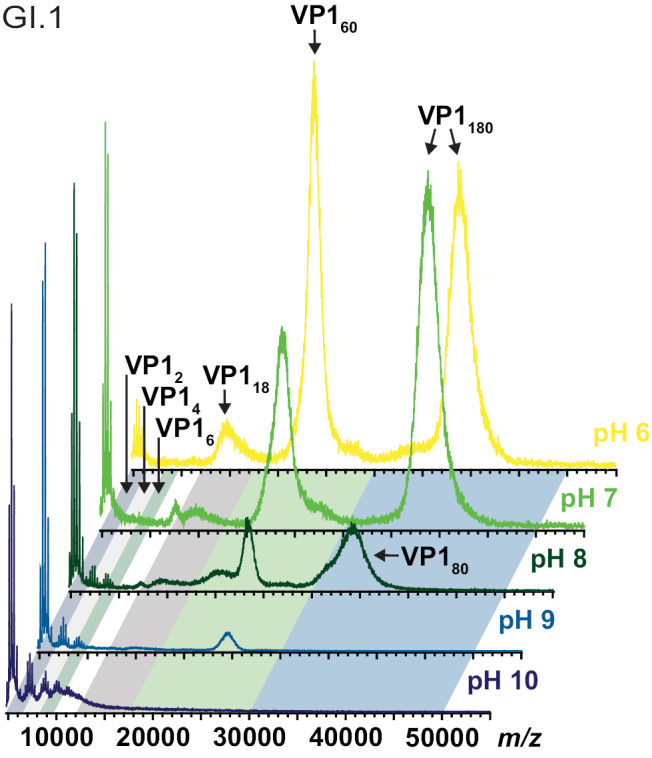
Norovirus GI.1 West Chester VLP stability monitored by native MS. Representative mass spectra of norovirus VLPs (10 *µ*M VP1) at pH values indicated (from top to bottom pH 6–10) in 250 mM ammonium acetate. Data are normalized to the highest peak.

**Figure 2.**
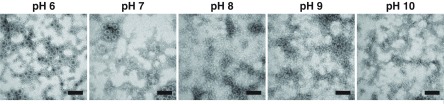

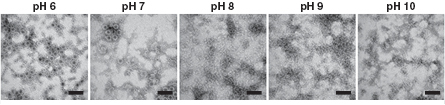

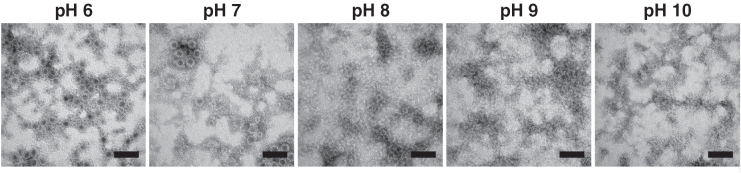
EM images of norovirus GI.1 West Chester VLPs. VLPs are in 250 mM ammonium acetate solutions with increasing pH from left to right. The bar represents 100 nm. All images are taken at the same magnification.

In order to compare the stability of VLPs within a genotype, native MS measurements in varying solution conditions were performed according to Shoemaker *et al* (2010). They observed the intact *T  =* 3 capsid, at neutral and near neutral pH, which disassembled upon alkaline treatment into VP1 oligomers. Whereas the prototypical GI.1 Norwalk virus was investigated previously, the GI.1 West Chester variant is studied here. Our assignment of ion distributions is analogous to the Norwalk results (Shoemaker *et al* 2010), since VP1 heterogeneity again precluded charge state resolution (figure S1). The broad ion distribution around *m*/*z* 40 000 indicates the presence of intact VLPs consisting of 180 copies of the VP1 protein (figure 1). The observed *m*/*z* is in line with the linear relation of charge state to the square root of the mass (Heck and van den Heuvel 2004) and is therefore assigned to *T*  =  3 particles. Following this scheme, further VP1 oligomers, namely VP1 80 mers and 60 mers (*T*  =  1), can be assigned in high ionic strength solutions (250 mM ammonium acetate) at pH ranging from 6–10 (figure 1). Intact *T*  =  3 is observed at pH 6 and pH 7. A shift of the corresponding ion distribution at pH 8 indicates that *T*  =  3 disassembles and instead VP1 80 mers form. A second dominant ion distribution is located around *m*/*z* 20 000. Ions in this *m*/*z* region can be assigned to the *T*  =  1 capsid. These ions are detected at pH 6 up to pH 9 and high ionic strength, although with strongly decreasing signals at alkaline pH. Several smaller oligomers are detected up to pH 8 between *m*/*z* 10 000–20 000 in line with disassembly of *T*  =  3 and reformation of 80 mer particles. The dominant signal around *m*/*z* 15 000 corresponds to VP1 18 mers already detected at pH 6. VP1 6 mers and 4 mers are observed in solutions at pH 7 and higher. VP1 dimers are present in all tested solutions with strongly increasing signals upon alkaline treatment. To further illustrate and confirm the different VP1 oligomers, the ammonium acetate VLP preparations were imaged in negative stain EM (figure 2). At pH 6 and pH 7, round structures with a diameter of about 40 nm corresponding to intact *T*  =  3 capsids can be found. Smaller particles with a diameter of about 20 nm are also seen in pH 6–8, likely the detected *T*  =  1 particles. Size estimation is affected by aggregation. However, a clear trend in line with the MS measurements is observed as bigger spherical structures are no longer visible at pH 9 and 10.

Notably, West Chester VP1 60 mers are present in ammonium acetate solutions from pH 6 up to pH 9, whereas 60 mers were only observed at pH 8 and pH 9 for the Norwalk virus despite comparable measurement conditions and very similar purification protocols. In both protocols a baculovirus expression system was used and VLPs were purified via CsCl gradient centrifugation. The *T*  =  1 particles with a diameter of approximately 21 nm are a known byproduct in VLP production and also found in stool samples (Taniguchi *et al* 1981, White *et al* 1997). Truncated VP1 proteins were described as a possible reason for formation of smaller particles (Huo *et al* 2015). The versions of West Chester VP1 observed in the low *m*/*z* region after particle disassembly show differences in methionine processing (Δ*m* ~ 270 Da per monomer) as was previously described for Norwalk VP1 (Shoemaker *et al* 2010). Therefore, the *T*  =  1 formation likely represents an intrinsic property of the VP1 protein. This is also in agreement with the observation of *T*  =  3 particles at neutral pH of the Norwalk virus S domain (Baclayon *et al* 2011). Nevertheless, it cannot be ruled out that small particles in stool samples are *T*  =  3 particles lacking the P domain or other aberrant assemblies. Furthermore, West Chester VLPs are more sensitive to alkaline treatment as they disassemble at even lower pH than the previously described Norwalk virus (Shoemaker *et al* 2010). Smaller oligomers, such as the VP1 18 mer, are also detected at lower pH values for the West Chester. Importantly like Norwalk, the West Chester virus retained the ability to reversibly assemble when diluted from a high pH solution into a low pH solution including reformation of *T*  =  1 particles (figure S2).

### 3.2. GII.17 Kawasaki norovirus shows vast stability at alkaline pH

**Figure 3.**
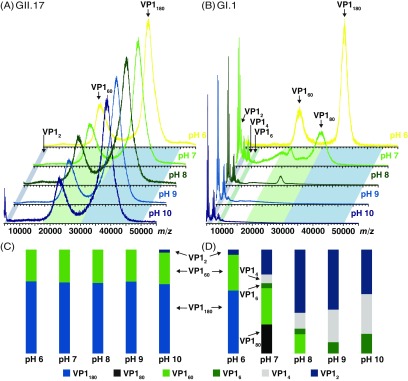

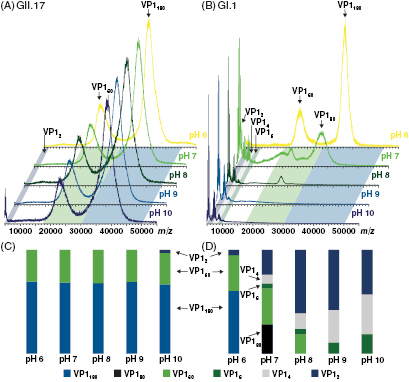

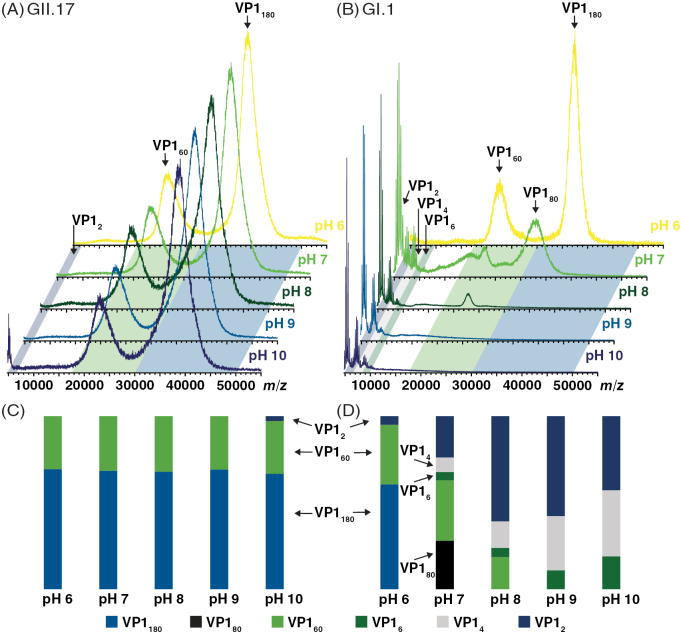
Comparison of norovirus VLP stability monitored by native MS at low ionic strength. Representative mass spectra of norovirus VLPs (10 *µ*M VP1) in 50 mM ammonium acetate at pH values indicated (from top to bottom pH 6–10) (A) mass spectra obtained for the GII.17 Kawasaki virus and (B) for the GI.1 West Chester virus. Data are normalized to the highest peak. Colored inlets indicate the respective *m*/*z* range, where intensities were summed for the respective VP1 oligomers. Due to overlapping distributions, the VP1 80 mer falls into the same *m*/*z* range as VP1 180 mers, the descriptor therefore changes at pH 7 in (D). The intensity fractions of oligomers are shown for GII.17 Kawasaki (C) and for GI.1 West Chester (D) viruses. Spectra for measurements in 250 mM ammonium acetate can be found in figure S3 of both variants.

Although the Norwalk virus is known as the prototype strain, GI noroviruses have been replaced by emerging GII noroviruses (Blanton *et al* 2006). It is therefore of interest, whether these still exhibit a similar stability profile as GI noroviruses, in other words whether particle stability is a conserved feature in noroviruses. Therefore, GII.17 Kawasaki VLPs are also analyzed with native MS (figure 3). Broad ion distributions corresponding to the *T*  =  3 and *T*  =  1 intact capsids are detected throughout the whole pH range (pH 6–10) both at low (50 mM ammonium acetate, figure 3) and high ionic strength (250 mM ammonium acetate, figure S3(A)). The ratios of the capsid sizes remain unchanged up to pH 9. At pH 10, mild disassembly is observed, as is evident from the arising dimer signal. Based on the intensity fractions, no preference for disassembly of either capsid form is observed. Disulfide bonds as cause of this extraordinary stabilization can be excluded, as no additional bands corresponding to oligomers are observed under non-reducing conditions in SDS-PAGE (figure S4).

**Figure 4.**
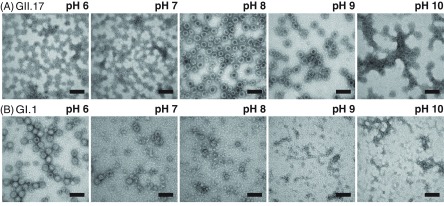

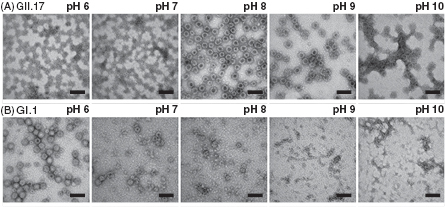

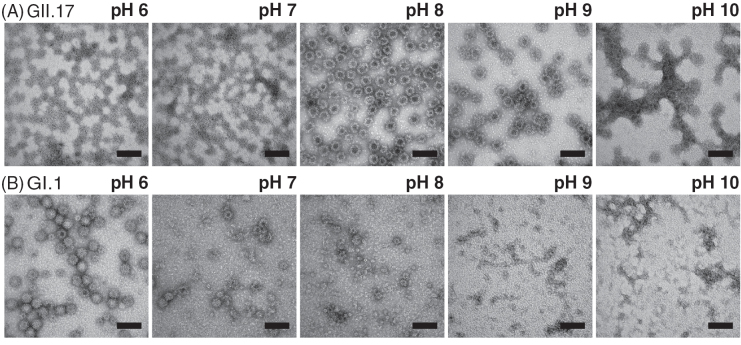
EM images of norovirus VLPs in 50 mM ammonium acetate. Images of VLPs of GII.17 Kawasaki virus (A) and of GI.1 West Chester virus (B) in increasing pH solutions from left to right (pH 6–10, respectively). The bar represents 100 nm. Images were taken at the same magnification.

Ions in the *m*/*z* range of 30 000–50 000 *m*/*z* assigned to *T*  =  3 signal cannot be detected above pH 7 for the GI.1 West Chester in 50 mM ammonium acetate (figure 3(B)). At pH 7 however, the signal already shifts to lower *m*/*z* as it does in 250 mM ammonium acetate, pH 8; therefore it likely corresponds to VP1 80 mers and not complete *T*  =  3 particles. At pH 9, also *T*  =  1 particles have disappeared. Thus, West Chester VLPs maintain a lower stability at lower ionic strength as was shown for Norwalk VLPs (Shoemaker *et al* 2010). Conversely, the Kawasaki VLPs show a more intense VP1 dimer signal at pH 10 at higher ionic strength indicative of decreased stability (figures 3(C) and S3(C)). VLP solutions were further imaged in negative stain EM (figure 4). Spherical structures with a diameter of about 40 nm can be found in all conditions for GII.17 Kawasaki VLPs. For GI.1 West Chester VLPs, the *T*  =  3 is most obvious at pH 6. Consistent with the findings at high ionic strength, several smaller West Chester VP1 oligomers arise with alkaline treatment albeit with less defined size (figures 1 and 3).

The mass spectra and intensity fractions are normalized and therefore do not include information on the absolute amount of assemblies or altered electrospray ionization due to pH induced changes. To further illustrate differences between the strains, absolute intensities for *m*/*z* ranges corresponding to *T*  =  3, *T*  =  1 and a combined lower *m*/*z* range (corresponding to VP1 oligomers between 4800 to 9600 *m*/*z* at 50 mM and 4800–17 500 *m*/*z* at 250 mM) are plotted (figure S5). The GII.17 ion signals for both capsid forms are increasing up to pH 9 until they drop again at pH 10 in 250 mM ammonium acetate. The ion counts for both forms are overall lower at 50 mM ammonium acetate but less affected by pH. Furthermore, the spray is more stable at low ionic strength as is evident from smaller error bars. In contrast, ion counts for GI.1 West Chester capsids are always lower but reproducible. High intensity counts can only be obtained for the signal corresponding to low mass VP1 oligomers upon VLP dissociation.

## 4. Discussion

The two human norovirus strains GI.1 West Chester and GII.17 Kawasaki show a completely different pattern when monitored in ammonium acetate solutions of increasing pH. The GI.1 West Chester virus behaves similar to Norwalk virus (Shoemaker *et al* 2010) and disassembles upon alkaline treatment into capsid protein oligomers. The alkaline sensitivity is furthermore dependent on ionic strength, as higher ionic strength favors higher-mass oligomers. In stark contrast, GII.17 Kawasaki VLPs exhibit immense stability throughout the tested pH range up to pH 10. Capsid destabilization is indicated only at pH 10 as VP1 dimer peaks arise in low abundance. For GII.17 Kawasaki VLPs, the influence of ionic strength is low but inversed compared to GI.1 West Chester and Norwalk viruses.

Previous studies using various techniques on norovirus VLP stability were able to show alkaline sensitivity for the Norwalk virus (Ausar *et al* 2006, Shoemaker *et al* 2010, da Silva *et al* 2011). Stability studies on noroviruses are of special interest, as these are foodborne and need to persist in various environmental settings. The influence of temperature, ionic strength and pH on the adhesion of VLPs to surfaces also revealed higher stability and more adhesion for GII.4 viruses than GI.1 (da Silva *et al* 2011, Samandoulgou *et al* 2015). However, the investigated pH range was limited. In 2010, Donath and coworkers investigated the norovirus genotype GII.7 with atomic force microscopy (AFM) in a pH range of 2–10 (Cuellar *et al* 2010). Despite belonging to GII genogroup, VLPs were found to be sensitive to alkaline treatment. GII.7 noroviruses were reported as slowly evolving genotypes with decreasing prevalence (Hoffmann *et al* 2012). Based on these and our own findings on GI.1 and emerging GII.17 viruses, it is tempting to speculate whether increased VLP stability could be related to prevalence. However, other causes cannot be excluded and genome containing viruses likely have altered stability profiles.

**Figure 5.**
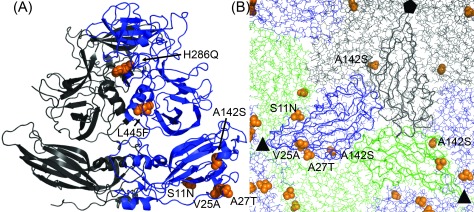

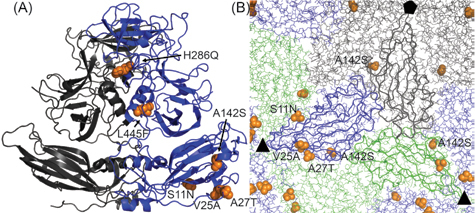

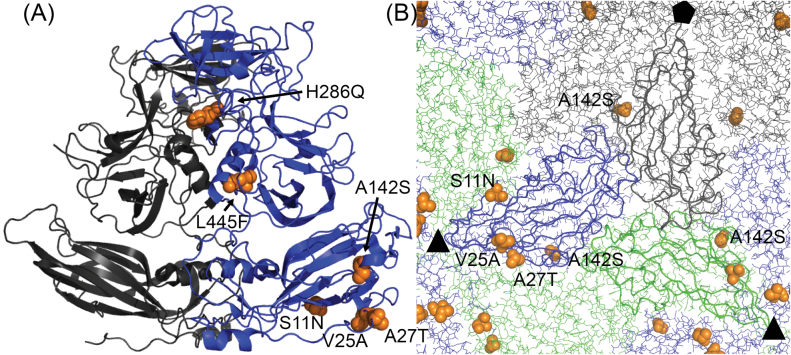
Norovirus GI.1 Norwalk and GI.1 West Chester capsid protein VP1 show six dissimilar substitutions. (A) Cartoon structure of a Norwalk VP1 dimer (PDB: 1IHM, residues 10–520) with quasi-equivalent monomers A (grey) and B (blue). Amino acids substituted for similar residues are not shown. Only the B subunit has a sufficiently resolved N-terminal arm to observe all four dissimilar amino acids in the S domain (bottom). The upper P domain only contains two major substitutions at the intra-dimer interface. All dissimilar residues are depicted as orange spheres and the substitution from Norwalk to West Chester is annotated. (B) S domains in the capsid context viewed from the interior with one subunit each (A—grey, B—blue, C—green) highlighted as ribbon. All substituted residues locate to the inter-dimer interface. Threefold and fivefold symmetry axes are labelled with black symbols.

A sequence alignment of VP1 of GI.1 Norwalk and West Chester virus reveals 13 substitutions (figure S6), with seven amino acids exchanged for similar residues. Four out of the six dissimilar residues are located in the S domain (N-terminal 225 amino acids); all of them are located at the dimer surface and potentially involved in inter-dimer contacts (figure 5). Substitutions for similar amino acids are exclusively found in the P domain together with two dissimilar residues close to the intra-dimer interface and therefore unlikely to cause the differences in assembly behavior. The P domain also contains the only substitution involving charged residues (H286Q), therefore altered electrostatic interactions are an unlikely cause of the observed changes. In light of the Norwalk S domain, which assembles into *T*  =  3 particles like the full length protein VP1 albeit with lower stability (Baclayon *et al* 2011), the amino acid exchanges in the S domain most likely cause the occurrence of the smaller West Chester *T*  =  1 at neutral pH. Which amino acids contribute to stability or alteration of capsid size requires further investigations by mutagenesis. Nevertheless, the quasi-equivalent *B* subunit shows a serine to asparagine substitution at residue 11 in the N-terminal arm, which directly interfaces with the *β*-sheet of subunit C. A/B and C/C dimers show a different arrangement of the S domain, bent and flat, respectively (Prasad *et al* 1999). The substitution at residue 11 may alter the capability of the VP1 protein to switch to the alternate C conformation and therefore also small particles can form at neutral pH. The GI.1 Norwalk and GII.17 Kawasaki sequences are 46% identical (figure S6). The substitutions are located across the entire sequence but with higher frequency in the P domain. However, in all three variants the pI remains similar (Norwalk virus pI 5.64, West Chester isolate pI 5.57 and Kawasaki isolate pI 5.24). Given the number of substitutions, it is impossible to link the increased stability to any specific residues. Interestingly, residue 11 is not substituted in Kawasaki virus but flanked by two substitutions, S10P and V12N, which could again be an explanation for the increased *T*  =  1 amount compared to Norwalk virus. Again, further studies are required to pinpoint the residues responsible for the observed stability and size differences.

This study demonstrates that native MS is the method of choice to investigate genotype and even isolate specific stability differences as it can resolve the different assembly states. The combination with negative stain EM confirmed the observed differences between norovirus-like particles of different genogroups. Notably, also isolates from the same genotype show distinct profiles and can pinpoint residues altering the assembly. The results are therefore informative for designing capsids with specific properties for nanotechnological applications (Worsdorfer *et al* 2011). It is of interest to test whether ligand binding to e.g. glycans alters the stability profiles, which would be indicative of structural changes. Furthermore, if stability differences persist at the virion level, mass determination in conjunction with determination of mechanical properties from AFM or similar methods could have potential as a diagnostic tool with the capability to distinguish specific isolates. Especially for rapidly evolving RNA viruses like noroviruses, this is a promising approach to identify new variants without the need for prior knowledge.

## Supplementary Material





## References

[cmaaa43bbib001] Ahmed S M, Hall A J, Robinson A E, Verhoef L, Premkumar P, Parashar U D, Koopmans M, Lopman B A (2014). Global prevalence of norovirus in cases of gastroenteritis: a systematic review and meta-analysis. Lancet Infect. Dis..

[cmaaa43bbib002] Ausar S F, Foubert T R, Hudson M H, Vedvick T S, Middaugh C R (2006). Conformational stability and disassembly of Norwalk virus-like particles. Effect of pH and temperature. J. Biol. Chem..

[cmaaa43bbib003] Baclayon M, Shoemaker G K, Uetrecht C, Crawford S E, Estes M K, Prasad B V, Heck A J, Wuite G J, Roos W H (2011). Prestress strengthens the shell of Norwalk virus nanoparticles. Nano Lett..

[cmaaa43bbib004] Blanton L H, Adams S M, Beard R S, Wei G, Bulens S N, Widdowson M-A, Glass R I, Monroe S S (2006). Molecular and epidemiologic trends of caliciviruses associated with outbreaks of acute gastroenteritis in the United States, 2000–2004. J. Infect. Dis..

[cmaaa43bbib005] Chan M C, Lee N, Hung T N, Kwok K, Cheung K, Tin E K, Lai R W, Nelson E A, Leung T F, Chan P K (2015). Rapid emergence and predominance of a broadly recognizing and fast-evolving norovirus GII.17 variant in late 2014. Nat. Commun..

[cmaaa43bbib006] Cuellar J L, Meinhoevel F, Hoehne M, Donath E (2010). Size and mechanical stability of norovirus capsids depend on pH: a nanoindentation study. J. Gen. Virol..

[cmaaa43bbib007] da Silva A K, Kavanagh O V, Estes M K, Elimelech M (2011). Adsorption and aggregation properties of norovirus GI and GII virus-like particles demonstrate differing responses to solution chemistry. Environ. Sci. Technol..

[cmaaa43bbib008] Eden J S, Tanaka M M, Boni M F, Rawlinson W D, White P A (2013). Recombination within the pandemic norovirus GII.4 lineage. J. Virol..

[cmaaa43bbib009] Hansman G S (2006). Genetic and antigenic diversity among noroviruses. J. Gen. Virol..

[cmaaa43bbib010] Hansman G S, Natori K, Oka T, Ogawa S, Tanaka K, Nagata N, Ushijima H, Takeda N, Katayama K (2005). Cross-reactivity among sapovirus recombinant capsid proteins. Arch. Virol..

[cmaaa43bbib011] Hansman G S, Saito H, Shibata C, Ishizuka S, Oseto M, Oka T, Takeda N (2007). Outbreak of gastroenteritis due to sapovirus. J. Clin. Microbiol..

[cmaaa43bbib012] Harrington P R, Lindesmith L, Yount B, Moe C L, Baric R S (2002). Binding of norwalk virus-like particles to ABH histo-blood group antigens is blocked by antisera from infected human volunteers or experimentally vaccinated mice. J. Virol..

[cmaaa43bbib013] Heck A J R, van den Heuvel R H H (2004). Investigation of intact protein complexes by mass spectrometry. Mass Spectrom. Rev..

[cmaaa43bbib014] Hedberg C W, Osterholm M T (1993). Outbreaks of food-borne and waterborne viral gastroenteritis. Clin. Microbiol. Rev..

[cmaaa43bbib015] Hoffmann D, Hutzenthaler M, Seebach J, Panning M, Umgelter A, Menzel H, Protzer U, Metzler D (2012). Norovirus GII.4 and GII.7 capsid sequences undergo positive selection in chronically infected patients. Infect. Genet. Evol..

[cmaaa43bbib016] Huo Y, Wan X, Wang Z, Meng S, Shen S (2015). Production of norovirus VLPs to size homogeneity. Virus Res..

[cmaaa43bbib017] Jiang X, Wang M, Graham D Y, Estes M K (1992). Expression, self-assembly, and antigenicity of the Norwalk virus capsid protein. J. Virol..

[cmaaa43bbib018] Jiang X, Wang M, Wang K, Estes M K (1993). Sequence and genomic organization of Norwalk virus. Virology.

[cmaaa43bbib019] Kageyama T, Shinohara M, Uchida K, Fukushi S, Hoshino F B, Kojima S, Takai R, Oka T, Takeda N, Katayama K (2004). Coexistence of multiple genotypes, including newly identified genotypes, in outbreaks of gastroenteritis due to Norovirus in Japan. J. Clin. Microbiol..

[cmaaa43bbib020] Kapikian A Z, Wyatt R G, Dolin R, Thornhill T S, Kalica A R, Chanock R M (1972). Visualization by immune electron microscopy of a 27 nm particle associated with acute infectious nonbacterial gastroenteritis. J. Virol..

[cmaaa43bbib021] Kaplan J E, Feldman R, Campbell D S, Lookabaugh C, Gary G W (1982). The frequency of a Norwalk-like pattern of illness in outbreaks of acute gastroenteritis. Am. J. Public Health.

[cmaaa43bbib022] Keifer D Z, Pierson E E, Jarrold M F (2017). Charge detection mass spectrometry: weighing heavier things. Analyst.

[cmaaa43bbib023] Lee C-C, Feng Y, Chen S-Y, Tsai C-N, Lai M-W, Chiu C-H (2015). Emerging Norovirus GII.17 in Taiwan. Clin. Infect. Dis..

[cmaaa43bbib024] Lorenzen K, Versluis C, Van Duijn E, Van Den Heuvel R H H, Heck A J R (2007). Optimizing macromolecular tandem mass spectrometry of large non-covalent complexes using heavy collision gases. Int. J. Mass Spectrom..

[cmaaa43bbib025] Mallagaray A, Lockhauserbaumer J, Hansman G, Uetrecht C, Peters T (2015). Attachment of norovirus to histo blood group antigens: a cooperative multistep process. Angew. Chem., Int. Ed. Engl..

[cmaaa43bbib026] Ozawa K, Oka T, Takeda N, Hansman G S (2007). Norovirus infections in symptomatic and asymptomatic food handlers in Japan. J. Clin. Microbiol..

[cmaaa43bbib027] Prasad B V, Hardy M E, Dokland T, Bella J, Rossmann M G, Estes M K (1999). X-ray crystallographic structure of the Norwalk virus capsid. Science.

[cmaaa43bbib028] Prasad B V, Rothnagel R, Jiang X, Estes M K (1994). Three-dimensional structure of baculovirus-expressed Norwalk virus capsids. J. Virol..

[cmaaa43bbib029] Samandoulgou I, Hammami R, Morales Rayas R, Fliss I, Jean J (2015). Stability of secondary and tertiary structures of virus-like particles representing noroviruses: effects of pH, ionic strength, and temperature and implications for adhesion to surfaces. Appl. Environ. Microbiol..

[cmaaa43bbib030] Shoemaker G K, Van Duijn E, Crawford S E, Uetrecht C, Baclayon M, Roos W H, Wuite G J, Estes M K, Prasad B V, Heck A J (2010). Norwalk virus assembly and stability monitored by mass spectrometry. Mol. Cell. Proteomics.

[cmaaa43bbib031] Singh B K, Koromyslova A, Hefele L, Gurth C, Hansman G S (2016). Structural evolution of the emerging 2014-2015 GII.17 noroviruses. J. Virol..

[cmaaa43bbib032] Tan M, Jiang X (2005). The p domain of norovirus capsid protein forms a subviral particle that binds to histo-blood group antigen receptors. J. Virol..

[cmaaa43bbib033] Taniguchi K, Urasawa S, Urasawa T (1981). Further studies of 35–40 nm virus-like particles associated with outbreaks of acute gastroenteritis. J. Med. Microbiol..

[cmaaa43bbib034] Teunis P F M, Moe C L, Liu P, Miller S E, Lindesmith L, Baric R S, Le Pendu J, Calderon R L (2008). Norwalk virus: how infectious is it?. J. Med. Virol..

[cmaaa43bbib035] Uetrecht C, Barbu I M, Shoemaker G K, Van Duijn E, Heck A J (2011). Interrogating viral capsid assembly with ion mobility-mass spectrometry. Nat. Chem..

[cmaaa43bbib036] Uetrecht C, Heck A J (2011). Modern biomolecular mass spectrometry and its role in studying virus structure, dynamics, and assembly. Angew. Chem., Int. Ed. Engl..

[cmaaa43bbib037] Van Den Heuvel R H (2006). Improving the performance of a quadrupole time-of-flight instrument for macromolecular mass spectrometry. Anal. Chem..

[cmaaa43bbib038] Vongpunsawad S, Venkataram Prasad B V, Estes M K (2013). Norwalk virus minor capsid protein VP2 associates within the VP1 shell domain. J. Virol..

[cmaaa43bbib039] Weiss V U (2015). Analysis of a common cold virus and its subviral particles by gas-phase electrophoretic mobility molecular analysis and native mass spectrometry. Anal. Chem..

[cmaaa43bbib040] White L J, Hardy M E, Estes M K (1997). Biochemical characterization of a smaller form of recombinant Norwalk virus capsids assembled in insect cells. J. Virol..

[cmaaa43bbib041] Worsdorfer B, Woycechowsky K J, Hilvert D (2011). Directed evolution of a protein container. Science.

[cmaaa43bbib042] Xi J, Graham D, Wang K, Estes M (1990). Norwalk virus genome cloning and characterization. Science.

[cmaaa43bbib043] Zhang X F (2015). An outbreak caused by GII.17 norovirus with a wide spectrum of HBGA-associated susceptibility. Sci. Rep..

